# Protective effect of quinacrine against glycerol-induced acute kidney injury in rats

**DOI:** 10.1186/s12882-017-0450-8

**Published:** 2017-01-28

**Authors:** Abdulrahman K. Al Asmari, Khalid Tariq Al Sadoon, Ali Ahmed Obaid, Deivakadatcham Yesunayagam, Mohammad Tariq

**Affiliations:** 1 Scientific Research Centre, Prince Sultan Military Medical City, Riyadh, Saudi Arabia; 2 Department of Urology, Prince Sultan Military Medical City, Riyadh, Saudi Arabia

**Keywords:** Glycerol, Acute kidney injury, Quinacrine, Phospholipase A2, Oxidative stress, Inflammation

## Abstract

**Background:**

Acute kidney injury (AKI) is a serious clinical problem with high rate of mortality and morbidity. Currently used prophylactic and therapeutic strategies to address AKI are limited and warrant further studies. In the present study an attempt was made to investigate the effect of quinacrine, a phospholipase A2 inhibitor against glycerol induced AKI in rats.

**Methods:**

Adult female Wistar rats were divided in to five groups. After 24 h of water deprivation rats in groups 3, 4 and 5 received an intraperitoneal injection of quinacrine (3 mg/kg, 10 mg/kg and 30 mg/kg of body weight respectively). Thirty minutes after the first injection of quinacrine animals in groups 3, 4 and 5 received an intramuscular injection of 25% glycerol (10 ml/kg of body weight). The animals in group 2 received 25% glycerol (10 ml/kg of body weight) only whereas rats in group 1 served as control . The quinacrine administration was continued once daily for three days, on the fourth day animals were sacrificed, blood and kidney were collected for various biochemical and histopathological studies.

**Results:**

Glycerol treatment produced significant renal structural abnormalities and functional impairment (increased urea and creatinine). Increase in myeloperoxidase (MPO) and malondialdehyde (MDA) clearly suggested the involvement of oxidative stress and neutrophilic activity following glycerol administration. Quinacrine dose dependently attenuated glycerol induced structural and functional changes in kidney.

**Conclusion:**

The reversal of glycerol induced AKI by quinacrine points towards a role of phospholipase A2 (PLA2) in the pathogenesis of renal injury. The result of this study suggests that quinacrine may offer an alternative mode of treatment for AKI.

## Background

Acute kidney injury, previously termed acute renal failure, is associated with increased mortality, prolonged hospital stay, and accelerated chronic kidney disease. Over the past few decades, dramatic rises in the incidences of AKI have been reported. Despite the reversibility of AKI in the patients who survive, mortality from AKI remains high (over 50%) [1]. In the recent years, the search for effective therapy to accelerate recovery and attempts to prevent AKI have attracted much attention. One of the most commonly used experimental models for studying AKI is the rat receiving a single intramuscular injection of glycerol, which induces rhabdomyolysis [2, 3]. Rhabdomyolysis-induced myoglobinuric renal failure accounts for about 10–40% of all cases of AKI [4]. Moreover during the time of war [5] and natural disaster [6] rhabdomyolysis induced myoglobinuric injury reached an epidemic state. Glycerol-induced AKI in rodents is mediated by renal ischemia and myoglobin nephrotoxicity [7, 8]. In glycerol-induced AKI, redox cycle, the myoglobin heme induces oxidative stress and lipid peroxidation of the proximal tubular cell, triggers the release of a series of mediators, including cytokines and chemokines, leading to leukocyte activation, resulting in tubular necrosis in the cortical area [9–12]. However the precise molecular mechanism of glycerol induced nephrotoxicity is still unclear. Some recent studies suggested that glycerol induced ischemic insult to the renal tissue may result in derangement of cellar phospholipid membranes, which may trigger a sequence of biochemical events leading to irreversible cell injury, presumably due to enhanced generation of oxygen derived free radicals and activation of tissue phospholipases [13, 14].

There is ample evidence which suggest that the critical events determining the course of AKI develop from damage to plasma and subcellular membranes [15]. Phospholipids besides providing the major structural framework for cell membranes [16] participate in the regulation of membrane enzyme activity, permeability and hormone activation [17–20]. Ischemic injury has been associated with depletion of major phospholipids and accumulation of phospholipid by-products in liver, heart and renal cortex [21–23]. The activation of membrane phospholipases, especially phospholipase PLA2 has been shown to play a pivotal role in the critical early events in the pathogenesis of ischemic cell injury [24]. Moreover increased PLA_2_ activity also leads to the release of arachidonic acid (AA) from membrane phospholipids [25] which is the precursor for the biosynthesis of vasoactive prostaglandins and leukotrienes [26]. A blockade of PLA_2_ could result in the suppression of these important classes of vasoactive lipid mediators and may offer an attractive tool to understand the pathophysiology of cytotoxic chemicals and to design novel agents that could attenuate or prevent tissue injury. Hence, the present investigation is an attempt to study effect of quinacrine, a potent inhibitor of PLA_2_ [27] on glycerol induced deleterious effects on renal structure and function in rats.

## Methods

### Animals

Animals were received from the Animal Facility Unit of Research Centre, Prince Sultan Military Medical City, Riyadh, Saudi Arabia. Forty adult female Wistar rats weighing 200 ± 10 g were housed in a 12 h dark/light cycle animals facility with controlled temperature and humidity. Food and water were given ad libitum throughout the study.

### Drugs

Quinacrine obtained from ICN Biochemical Inc, USA. Glycerol 99%, Pentobarbital sodium and other chemical used for biochemical assays were purchased from Sigma Chemical Co., St. Louis., MO., USA.

### Experimental design

The protocol suggested by Midhun et al. [28] was followed to study the effect of quinacrine on glycerol induced acute renal injury. The detail of groups, treatments and sacrificing protocol were tabulated in Table 1. The rats were randomized and divided into five groups of eight animals each. The number of animals in each group were calculated according to the method described by Charan and Kanthria et al. [29] which is based on the value “Effect size (difference between mean of the groups) standard deviation taken from our earlier studies along with a power level 80% and significance level of 5%”.

**Table 1:** Drug treatment protocol

Groups	Day-0	Day-1	Day-2	Day-3	Day-4
Control	WD	saline	saline	saline	Sacrificed
GLY 25% (GLY)	WD	GLY	saline	saline	Sacrificed
QRN 3 mg/kg + GLY	WD	QRN + GLY	QRN	QRN	Sacrificed
QRN 10 mg/kg + GLY	WD	QRN + GLY	QRN	QRN	Sacrificed
QRN 30 mg/kg + GLY	WD	QRN + GLY	QRN	QRN	Sacrificed

*Abbreviations*: *WD* water deprived, QRN quinacrine, *GLY* glycerol 25%

After being water deprived for 24 h, animals from group 1 and 2 received intraperitoneal injection of normal saline (2 ml/kg of body weight), whereas group 3,4 and 5 received intraperitoneal injection of quinacrine at the dose of 3 mg/kg, 10 mg/kg and 30 mg/kg of body weight. After 30 min of first injection of quinacrine, rats in group 2,3,4 and 5 received a single intramuscular injection of 25% glycerol (10 ml/kg body weight). Quinacrine injection (prepared freshly) was continued once daily for three days. Body weight, food and fluid intake were recorded daily at specific time. Twenty four hours after last injection of quinacrine all the rats were sacrificed under general anesthesia using intraperitoneal injection of pentobarbital sodium (150 mg/kg ), blood was collected through heart puncture. The left side kidney was excised immediately, weighed, divided into four parts and stored in −70 °C for biochemical analysis. The right kidney was fixed in 10% neutral buffered formalin for histological studies.

### Serum biochemical analysis

All blood samples were allowed to clot at ambient temperature and centrifuged (3000 rpm for 10 min) to harvest the serum. Serum biochemical parameters of blood urea nitrogen (BUN), creatinine (Scr) calcium (Ca2+), magnesium, sodium, potassium and phosphorus levels were measured spectrophotometrically (APEL PD-303S Japan) using the commercially available kits BUN (REF-020), Scr (REF-033 K), calcium (REF-022), magnesium (REF-050), sodium (REF-054 K), potassium (REF-051), phosphorus (REF-046) from United Diagnostics Industry, Riyadh, Saudi Arabia.

### Determination of myeloperoxidase

The level of neutrophil derive enzyme MPO activity in kidney was measured according to the methods of Barone et al. [30]. The kidney tissue was homogenized (1:20 wt/vol) in 5 mM potassium phosphate buffer (pH 6.0). The homogenate was centrifuged at 17000 g for 15 min. at 4 °C. After discarding the supernatant the pellet was extracted with 0.5% hexadecyltrimethylammonium bromide in 50 mM potassium phosphate buffer (pH 6.0). The sample was subjected to three freeze-thaw cycles, with sonication (10 s, 25 °C) between cycles. After sonication, the samples were incubated at 4 °C for 2 min and again centrifuged at 12500 g (15 min, 4 °C). MPO activity in the supernatant was assayed by mixing 0.1 ml of supernatant with 2.9 ml of 50 mM potassium phosphate buffer (pH 6.0) containing 0.167 mg/mL o-dianasidinedihydrochloride and 0.0005% hydrogen peroxide. The change in absorbance at 460 nm was measured for 3 min with an UV spectrophotometer (Shimadzu, UV-160A, Japan).

### Determination lipidperoxidation

The level of thiobarbituricacid reactive substances (TBARS) as a method of lipid peroxidation in kidney tissue was measured according to the method described by Ohkawa et al. [31]. Approximately 0.5 g of kidney tissue was homogenized in 1.15% cold KCl. After centrifugation at 3000 g for 5 min, an aliquot of supernatant was mixed with 2 ml of reaction mixture (containing 15% trichloroacetic acid and 0.375% thiobarbituric acid solution in 0.25 N HCl) and heated for 5 min in a boiling water bath. The tubes were cooled at room temperature and centrifuged at 1000 g for 10 min. The absorbance of supernatant was read at 535 nm against a blank that contained all reagents except homogenate. Tissue lipid peroxide levels were calculated as nanomoles of MDA, tetramethoxy propane was used as standard.

### Histopathological evaluation

After recording the kidney weight and morphological examination, the kidney was fixed immediately in 10% formalin, embedded in paraffin, sectioned at 3 μm thickness and were stained with haematoxylin and eosin. The extent of tubular injury, dilatation, vacuolation and necrosis were evaluated semi-quantitatively. Briefly, the extent of tissue damages was graded from 0 to 4 according to the severity of, tubular dilatation, tubular vacuolation and tubular cell necrosis. The scoring system was as follows: 0 = no change in the tubules; 1 = < 25% of tubular injury (mild); 2 = 25% to 50% of tubular involvement (moderate); 3 = 50% to 75% of tubules showing characteristic change (severe) and 4 = more than 75% of tubular damage (very severe). Fifty fields were counted from each slide. All the assessments were done in a blinded fashion.

### Kidney weight-to-body weight ratio

At the time of sacrifice the body weights and the kidney weights of rats were recorded. The kidney weight to body weight (KW/BW) ratio was calculated by simple arithmetic calculation of the kidney weight divided by body weight and then converted to percent.

### Statistical analysis

All results are presented as Mean ± S.E.M. Statistical significance was determined by one way analysis of variance (ANOVA) followed by Dunnett’s test. In all cases *P* < 0.05 was considered statistical significant.

## Results

### Effect of glycerol and quinacrine on blood urea nitrogen and serum creatinine

**Fig. 1:**
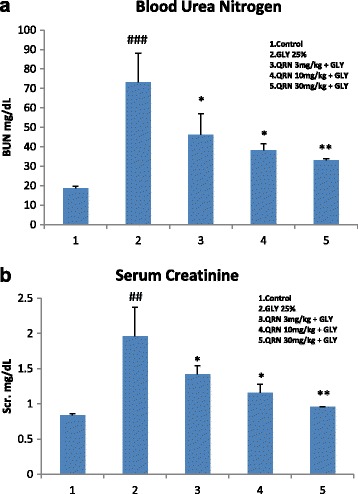
Blood urea nitrogen and serum creatinine levels. **a**. The effect of quinacrine on glycerol induced changes in BUN. Glycerol produced a significant (*P* < 0.001) increase in BUN as compare to control group (###). Treatment of rats with quinacrine in the doses of 3 mg/kg (*P* < 0.05), 10 mg/kg (*P* < 0.05) and 30 mg/kg (*P* < 0.01) significantly attenuated glycerol induced rise in BUN (* values compared to glycerol treated group). **b**. The effect of quinacrine on glycerol induced changes in Scr. Glycerol produced a significant (*P* < 0.01) increase in Scr. as compare to control group (##). Treatment of rats with quinacrine in the doses of 3 mg/kg (*P* < 0.05), 10 mg/kg (*P* < 0.05) and 30 mg/kg (*P* < 0.01) significantly attenuated glycerol induced rise in Scr. (* values compared to glycerol treated group)

The BUN and Scr. the two principal clusters of renal function biomarkers were recorded in this study. There was a highly significant increase (*P* < 0.001) in serum BUN (73.17 ± 14.9 mg/dL) in glycerol treated rats as compared to control group (18.80 ± 1.326 mg/dL ). The level of serum BUN following low (46.1 ± 10.7 mg/dL, *P* < 0.05), medium (38.1 ± 3.5 mg/dL, *P* < 0.05), and high (33.0 ± 0.7 mg/dL, *P* < 0.01) dose treatment of quinacrine showed a significant attenuation of glycerol induced increase in BUN level (Fig. 1a).

Treatment of rats with glycerol also produced a significant increase (*P* < 0.01) in Scr. (1.9 ± 0.4 mg/dL) as compared to control group (0.83 ± 0.024 mg/dL) in 72 h. The level of Scr. following low (1.4 ± 0.12 mg/dL, *P* < 0.05), medium (1.15 ± 0.12 mg/dL, *P* < 0.05), and high (0.95 ± 0.005 mg/dL, *P* < 0.01) doses of quinacrine showed a dose dependent and significant attenuation of glycerol induced increase in Scr. levels (Fig. 1b).

### Effect of glycerol and quinacrine on serum electrolytes and phosphorus

Acute kidney injury is associated with a significant electrolyte and acid–base disturbance. Even mild electrolyte disorders may be associated with highly significant morbidity and mortality. There was a significant (*P* < 0.01) increase in serum calcium (9.7 ± 0.16 mg/dL ) in glycerol treated rats as compared to control group (9.02 ± 0.123 mg/dL ). The serum level of calcium following treatment with low (9.27 ± 0.164 mg/dL, *P* < 0.05 ), medium (9.12 ± 0.104 mg/dL, *P* < 0.001 ), and high (9.00 ± 0.004 mg/dL, *P* < 0.001) doses of quinacrine showed a highly significant attenuation of glycerol induced increase in serum calcium level (Table 2).

**Table 2:** Effect of quinacrine on glycerol-induced AKI; level of serum electrolytes and phosphorus

Groups	Calcium mg/dL	Magnesium mg/dL	Sodium mmol/L	Potassium mmol/L	Phosphorus mg/dL
Control	9.21 ± 0.123	1.76 ± 0.111	140 ± 4.4	3.96 ± 0.25	5.98 ± 0.119
GLY 25%	9.73 ± 0.164 ##	3.23 ± 0.237###	135 ± 3.7	5.46 ± 0.302#	7.53 ± 0.588#
QRN 3 mg/kg + GLY	9.27 ± 0.164 *	2.45 ± 0.206*	136 ± 4.0	5.15 ± 0.302	6.68 ± 0.706
QRN 10 mg/kg + GLY	9.12 ± 0.104***	2.33 ± 0.155**	143 ± 6.8	4.2 ± 0.401	5.86 ± 0.676
QRN 30 mg/kg + GLY	9.00 ± 0.004***	2.03 ± 0.113***	151 ± 5.6*	3.85 ± 0.241**	4.8 ± 0.311***

Single intramuscular glycerol injection significantly increase the level of serum calcium, magnesium, potassium, phosphorus and lowered the level of sodium, whereas quinacrine treatment attenuate this alteration in serum electrolytes. Data expressed as mean ± SE. # *P* < 0.05, ## *P* < 0.01and ### *P* < 0.001 as compare with control group. *P < 0.05, **P < 0.01 and *** *P* < 0.001 as compare to glycerol treated group

Treatment of rats with glycerol produced a highly significant (*P* < 0.001) increase serum magnesium level (3.23 ± 0.237 mg/dL) as compared to control rats (1.76 ± 0.111 mg/dL). The serum magnesium levels following low (2.45 ± 0.206 mg/dL, *P* < 0.05), medium (2.33 ± 0.155 mg/dL, *P* < 0.01), and high (2.03 ± 0.113 mg/dL, *P* < 0.001) doses of quinacrine showed a highly significant reduction of glycerol induced increase in magnesium level (Table 2).

There was a decrease (135 ± 3.7 mmol/L ) in serum sodium level of glycerol treated rats as compared to rats in control (140 ± 4.4 mmol/L ) group. The glycerol induced decrease in sodium level was attenuated by low (136 ± 4.0 mmol/L ), medium (143 ± 6.8 mmol/L), and high (151 ± 5.6 mmol/L *P* < 0.05) doses of quinacrine (Table 2).

Glycerol treatment significant increased (*P* < 0.05) serum potassium level (5.46 ± 0.302 mmol/L) as compared to control rats (3.96 ± 0.25 mmol/L). The potassium levels in the rat treated with low (5.15 ± 0.302 mmol/L), medium (4.2 ± 0.401 mmol/L) and high (3.85 ± 0.241 mmol/L, *P* < 0.01) doses of quinacrine showed a reversal of glycerol induced increase in potassium level (Table 2).

Glycerol produced a significant (*P* < 0.05) increase in serum phosphorus level (7.53 ± 0.588 mg/dL) as compared to control group (3.96 ± 0.25 mg/dL ). The serum level of phosphorus in the rat treated with low (6.68 ± 0.706 mg/dL), medium (5.86 ± 0.676 mg/dL ), and high (4.8 ± 0.311 mg/dL, *P* < 0.001) doses of quinacrine showed a dose dependent attenuation of glycerol induced increase in serum phosphorus level (Table 2).

### Renal myeloperoxidase and malodialdehyde levels

**Fig. 2:**
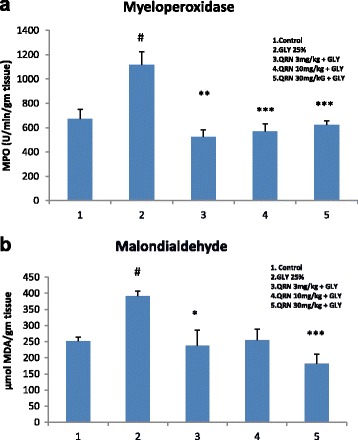
Myeloperoxidase and malondialdehyde in kidney. **a** The effect of quinacrine on glycerol induced changes kidney MPO activity. Glycerol produced a significant (*P* < 0.01) increase in kidney MPO activity as compare to control group (#). Treatment of rats with quinacrine in the doses of 3 mg/kg, 10 mg/kg (*P* < 0.001) and 30 mg/kg (*P* < 0.001) significantly attenuated glycerol induced rise in kidney MPO activity Scr. (* values compared to glycerol treated group). **b** The effect of quinacrine on glycerol induced changes kidney MDA level. Glycerol produced a significant (*P* < 0.01) increase in kidney MDA level as compare to control group (#). Treatment of rats with quinacrine in the doses of 3 mg/kg (*P* < 0.05), 10 mg/kg and 30 mg/kg (*P* < 0.001) significantly attenuated glycerol induced rise in kidney MDA level. (* values compared to glycerol treated group)

The neutrophil derived enzyme MPO is considered an important pathophysiogic factor in progression of renal disease. The tissue MPO activity was significantly (*P* < 0.05) higher (1118.11 ± 106.5 U/min/gm tissue) in glycerol treated rats as compared to control group (673.8 ± 77.7 U/min/gm tissue). The MPO activity in the rat treated with low (523.4 ± 60.8 U/min/gm tissue, *P* < 0.01), medium (571.04 ± 60.5 U/min/gm tissue, *P* < 0.001), and high (623.75 ± 30.5 U/min/gm tissue, *P* < 0.001) doses of quinacrine showed a significant attenuation of glycerol induced increase in MPO activity in kidney tissues (Fig. 2a).

Ischemia and chemical induced renal injury is associated with significant increase in MDA level. Treatment of rats with glycerol produced a significant (*P* < 0.05) increase in tissue MDA levels (391.4 ± 14.8 μmol) as compared to control rats (251.5 ± 12 μmol). The MDA level of rat kidney treated with low (237.4 ± 49.2 μmol, *P* < 0.05), medium (254.7 ± 34.4 μmol ) and high (181 ± 30.1 μmol, *P* < 0.001) doses of quinacrine showed a significant attenuation of glycerol induced increase in MDA level in kidney tissues (Fig. 2b).

### Kidney weight to body weight ratio

There was a significant (*P* < 0.05) increase in KW (1.481 ± 0.041gm ) in glycerol treated rats as compared to control rats (0.651 ± 0.020 gm). The KW in the rats treated with low (1.302 ± 0.067 gm, *P* < 0.05), medium (1.293 ± 0.047 gm, *P* < 0.01), and high (1.017 ± 0.059 gm, *P* < 0.001) doses of quinacrine showed a significant attenuation of glycerol induced increase in KW (Table 3).

**Table 3:** Effect of quinacrine on glycerol induced body weight, kidney weight and kidney weight to body weight ratio

	Body weight (gm)	Kidney weight (gm)	KW /BW ratio
Control	209.5 ± 2.34	0.651 ± 0.020	0.309 ± 0.010
GLY 25%	196.8 ± 3.28	1.481 ± 0.041#	0.755 ± 0.031#
QRN 3 mg/kg + GLY	193.6 ± 3.17	1.302 ± 0.067*	0.694 ± 0.036
QRN 10 mg/kg + GLY	195.5 ± 2.86	1.293 ± 0.047**	0.634 ± 0.038*
QRN 30 mg/kg + GLY	198.6 ± 3.01	1.017 ± 0.059***	0.525 ± 0.029***

Effect of quinacrine on glycerol induced changes in kidney weight and body weight. The body weight was not change in all the treated groups but the kidney weight and KW/BW ratio significantly increased in glycerol treated groups. Quinacrine treatment showed a significant and dose dependent attenuation of glycerol induced increase in KW and KW/BW ratio. Data expressed as mean ± SE. # *P* < 0.001 as compare with control group and *P < 0.05, ** *P* < 0.01 and *** *P* < 0.001 as compared with Glycerol treated group

Similarly treatment of rats with glycerol produced a significant (*P* < 0.05) increase in KW/BW ratio (0.755 ± 0.031) as compared to control rats (0.309 ± 0.010). The KW/BW ratio in low (0.694 ± 0.036), medium (0.634 ± 0.038, *P* < 0.05), and high (0.525 ± 0.029, *P* < 0.001) dose of quinacrine treated rats showed a significant attenuation of KW/BW ratio (Table 3).

### Histological findings

The light microscopic findings of the kidneys of the treated rats are given in Table 4. The groups injected with glycerol showed widespread damage both in cortex and medullar region of the kidney. The microscopic changes include; the loss of microvilli, tubular dilatation and vacuolation, tubular necrotic lysis and cellular micro debris into the tubular lumen. Severe tubular damage were observed mainly in the distal end of the kidney, whereas inner medulla showed mainly tubular dilatation and cell debris.

**Table 4:** Effect of Quinacrine on glycerol induced histopathological changes in kidney (higher scores indicate severe injury)

Groups	Tubular dilatation	Tubular voculation	Tubular necrosis
Control	0	0	0
Glycerol25%	3.37 ± 0.18###	1.43 ± 0.290#	0.875 ± 0.182##
QRN 3 mg/kg + GLY	2.50 ± 0.25*	1.25 ± 0.163	0.812 ± 0.209
QRN 10 mg/kg + GLY	1.87 ± 0.27**	0.81 ± 0.209	0.375 ± 0.125*
QRN 30 mg/kg + GLY	1.37 ± 1**	0.62 ± 0.081*	0.125 ± 0.125**

Effect of quinacrine on glycerol-induced AKI.. Glycerol treated rats developed significant histological changes in kidney tubules. Data expressed in mean ± SE. # *P* < 0.05, ## *P* < 0.01 and ### *P* < 0.001 as compare to control group; *P < 0.05, and ** *P* < 0.001 as compare to glycerol treated group (G.3)

**Fig. 3:**
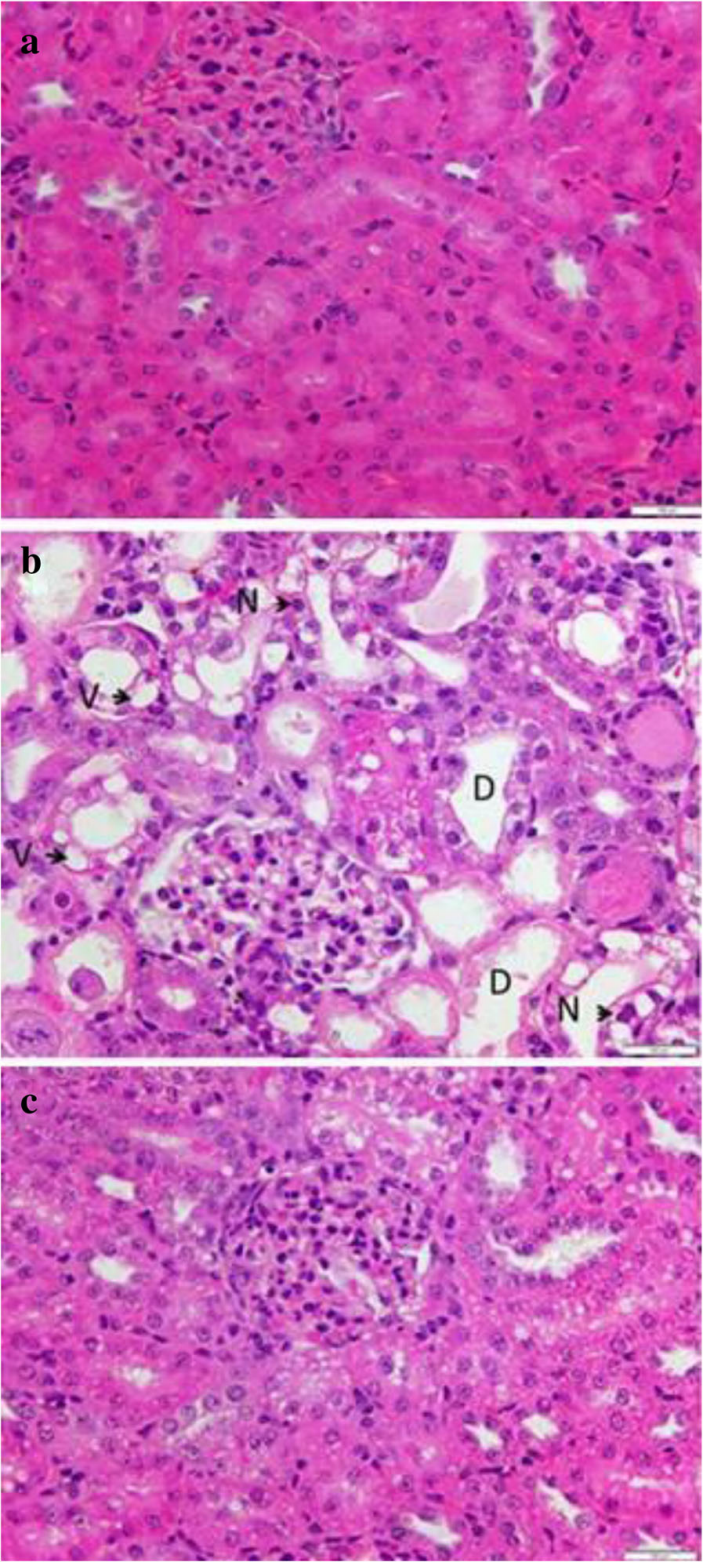
Histological appearance of kidney in control and treated rat. Representative renal histopathology using hematoxylin and eosin staining of sections presented with magnification x200. **a** Kidney section of control group, **b** Kidney section of glycerol treated group, **c** Kidney section of quinacrine and glycerol treated group. Microscopic changes include tubular dilatation (D), vacuolation (V) and necrosis (N)

Concomitant treatment with quinacrine significantly reversed glycerol induced histopathological changes in kidney (Fig. 3).

## Discussion

The results of this study demonstrated that the treatment of animals with glycerol produced a significant increase in serum BUN and Scr (Fig. 1) suggesting the functional impairment of kidneys. Glycerol treatment also resulted in development of edema and enlargement of kidney which was evidence from increase in kidney weight/body weight ratio confirming a significant toxic insult to the renal tissue. Our histopathological studies showed significant structural changes, including tubular dilatation, vacuolation, necrosis and cellular debris in the kidneys of glycerol treated rats. Similar biochemical and histopathological changes have been reported earlier following glycerol treatment [32, 33]. The treatment of rats with quinacrine dose dependently attenuated glycerol induced renal toxicity (Fig. 1 & Table 4). Our earlier studies showed highly significant protective effect of quinacrine against cyclosporine induced renal toxicity [34], ethanol induced gastric mucosal injury [35] and1-methyl-4-phenyl-1,2,3,6-tetrahydropyridine (MPTP) induced neuronal injury [36]. Quinacrine has also been shown to exert significant protective effect against ischemia induced myocardial [37] and cerebral [38, 39] injury.

Alteration of phospholipid metabolism during the renal ischemic injury is well documented [23, 40]. Glycerol induced ischemic insult results in derangement of cellular phospholipid membrane of renal tissue [12, 41]. PLA2 enzymes catalyze the hydrolysis of the 2-acyl bond of 3-n-phosphoglycerides, resulting in release of fatty acid from the second carbon group of glycerol. This enzyme specifically recognizes the sn-2 acyl bond of phospholipids and catalytically hydrolyzes the bond releasing arachidonic acid (AA) and lysophospholipids [42]. Free AA is the precursor of the vasoactive eicosanoids including prostaglandins [43]. Treatment with glycerol is known to significantly disturb the equilibrium between the AA metabolites including vasodilator prostacyclin (PGI2) and vasoconstrictor thromboxane (TxA2) in favor of TxA2 causing impairment of renal blood flow resulting in ischemic injury [44–46]. Quinacrine, a widely used inhibitor of lipolytic enzyme PLA2 has been shown to dose-dependently inhibit lipolytic release of arachidonate and generation of eicosanoids from kidney medulla [47–49]. Besides being a non-selective PLA2 inhibitor quinacrine has also been shown to possess significant cyclooxygenase inhibitory activity [50]. Thus the attenuation of glycerol induced renal injury by quinacrine may be attributed to inhibition of PLA2 induced lipolytic activity and restoration of physiological balance of the vasoactive eicosanoids in renal tissue resulting in stabilization of cell membrane.

The mechanism of glycerol induced renal injury is not entirely clear, a host of inflammatory mediators and cell mediated immune responses are believed to be involved in pathophysiology of AKI [12, 51]. We observed a significant increase in neutrophil-derived enzyme, MPO in the kidney of glycerol treated rats (Fig. 2) suggesting a robust neutrophil activity in the tissue [52]. Bolisetty and Agarwal [53] showed that neutrophil accumulate in kidney following ischemic insult due to their transmigration into the interstitium. Alteration of epithelial and endothelial cell integrity by neutrophils leads to kidney injury. Takasaki et al. [54] suggested that neutrophil cause kidney damage through the excessive release of oxygen radicals and proteases. In this study we observed a significant attenuation of MPO activity in the kidney tissue of quinacrine treated rats (Fig. 2). The exact mechanism by which quinacrine may reverse neutrophil mediated renal injury is not fully understood. Daniel et al. [55] reported a significant inhibition of neutrophil mediated superoxide generation and AA release by quinacrine. Earlier, anti-PLA2 antibodies have been shown to significantly suppress the neutrophil activity [56]. Beside affecting innate and adaptive immunity neutrophils are well recognized as one of the major player during inflammatory damage to the tissues [57]. Korrapati et al. [29] reported a significant increase in kidney NF-kB at 24 to 48 h after glycerol administration in rats. The available data clearly suggest that NF-kB and the major tumor suppressor P53 work in tandem in the pathogenesis of AKI [58, 59]. While NF-kB is a potent inflammatory mediator and plays a major role in the synthesis of pro-inflammatory cytokines and chemokines [60], the anti-inflammatory effect of P53 seems to be universal [59]. P53 has been shown to mitigate inflammation and exerts nephroprotective effect by several earlier investigators [61, 62]. Quinacrine and its derivatives have been shown to suppress NF-kB and increased P53 protein by causing chromatin trapping of the FACT (facilitates chromatin transcription) complex [63, 64]. Moreover quinacrine has been shown to inhibit histamine methyltransferase, a major enzyme responsible for catabolizing histamine, resulting in increased histamine level in kidney and other tissues [65]. Histamine participates in regulation of wide variety of pathophysiological events including vasomotor actively and inflammatory responses. Histamine infusion directly in renal artery decreases renal vascular resistance and increased blood flow through its action on H1 and H2 receptors [66]. Histamine through H1 receptors augment inflammatory responses [67]; whereas through H2 receptors it suppress inflammation by reducing inflammatory cytokines and chemokines [68, 69]. The ability of quinacrine to activate P53 and to inhibit NF-kB and histamine methyltrasferase may contribute to its nephroprotective activity [70, 71].

The result of this study showed a significant increase in kidney MDA levels of the rats treated with glycerol (Fig. 2). Malondialdehyde is a highly reactive molecule and one of the most reliable marker of oxidative stress. Glycerol-induced AKI has been shown to increase the generation of reactive oxygen species (ROS) and/or depletion of antioxidant defense system [64]. Kidney is an organ highly vulnerable to oxidative stress induced tissue injury, likely due to the abundance of long-chain polyunsaturated fatty acids in the composition of renal lipids [72]. Treatment of the rats with quinacrine significantly attenuated glycerol induced increase in kidney MDA levels (Fig. 2). Turnbull et al. [73] showed highly significant antioxidant activity of quinacrine. Inhibition of MDA production by quinacrine has been attributed to its ability to protect unsaturated fatty acids from lipid peroxidation by binding to membrane phospholipids [74]. Moreover Fujmoto et al. [75] suggested that lipid peroxidation is closely associated with prostaglandin generating system in kidney, especially at the AA (substrate) and cyclic endoperoxide level during the synthesis of prostaglandins. Quinacrine might decrease the production of MDA from endoperoxides by inhibiting the release of AA which is generated by phospholipid cell membrane under the influence of phospholipase enzymes. Besides inhibiting the oxidative stress and PLA2 activity, the nephroprotective effect of quinacrine may partly be attributed to the improvement of microcirculation as a result of its direct vasodilator activity [76, 77].

Treatment of rats with glycerol produced a significant increase in serum Ca2+ levels, whereas quinacrine dose dependently attenuated glycerol induced hypercalcemia (Table 2). Our findings support the earlier investigators who also observed high serum Ca2+ levels in glycerol treated rats [78, 79]. A key role of Ca2+ in cell injury has long been recognized. The lethal cell injury develops in a tissue due to mitochondrial accumulation and sequestration Ca2+. The major mechanisms by which Ca2+ promotes cell injury include activation of phospholipases, endonucleases, proteases and protein kinases direct and indirect effects on mitochondrial membrane permeabil and effects on contractile and cytoskeletal structures and functions [80]. Although our study is limited due to absence of tissue Ca2+ levels, a significant increase serum Ca2+ observed in this study (Table 2.) may be attributed to hypovolemia, metabolic acidosis [81] and compromised tubular fluid dynamics resulting in electrolyte imbalance following glycerol administration [82]. Reversal of toxin induced alteration in Ca2+ with a variety of agents has been beneficial in ameliorating the degree of cell injury in a number of experimental settings [83, 84].

In contrast to hypercalcemia, we observed a decrease in serum sodium (Na+) in glycerol treated rats. Our findings are in agreement with earlier investigators who reported a significant decrease in serum Na + in glycerol induced rhabdomyolysis in rats [85, 86]. The important role of Na + in the pathogenesis of glycerol induced renal injury is evident from the findings of numerous earlier studies. Infusion of Na + (150 mmol/L) protected animal against glycerol induced renal injury [87], whereas sodium restriction has been shown to aggravate glycerol induced acute renal failure [88]. Park et al. [72] showed that glycerol induced renal failure in rats was associated with significant increase in fractional excretion of sodium. Treatment of rats with quinacrine attenuated glycerol induced change in Na + and Ca2+ levels (Table 2). Glycerol induced kidney injury is associated with ischemic insult to renal tissue resulting in activation of PLA2 enzymes [89]. Phospholipase A2 and its metabolic products inducing AA and prostaglandins have been implicated in regulation of ion trafficking through the cell membranes [90]. Besides reversing PLA2 induced alteration of ion channels quinacrine has been shown to directly modulate ion channels in a selective manner [91, 92]. The result of this study clearly suggest that restoration of ion homeostasis may help in preventing renal injury.

There are some limitation in this study, we used serum creatinine and blood urea nitrogen as the biochemical marker of renal function, which were supported by our histopathological studies of kidney tissue. However urine analysis based studies including urine volume, urine creatinine and creatinine clearance tests could have strengthen our claims of attenuation of glycerol induced renal impairment by quinacrine . Moreover our claim that nephroprotective effect of quinacrine may be attributed to its universally known PLA2 inhibiting activity could have been further substantiated by measuring PLA2 activity and arachidonic acid metabolites in kidney tissue. Further studies are warranted to gain more insight in the mechanism of nephroprotective action of quinacrine.

## Conclusion

In conclusion, the result of this study suggest a significant role of oxidative stress, proinflammatory myeloperoxidase and electrolyte imbalance in the pathogenesis of glycerol induced renal injury. Treatment of rats with quinacrine produced a highly significant and dose dependent nephroprotection against glycerol induced renal injury. The ability of quinacrine to mitigate oxidative stress suppress inflammatory mediators and to maintain electrolyte hemostasis makes it potential candidate for therapeutic exploitation for the treatment of drug/chemical induced renal injury.

## Abbreviations

AA: Arachidonic acid
AKI: Acute kidney injury
ANOVA: One way analysis of variance
BUN: Blood urea nitrogen
BW: Body weight
Ca2+: Calcium
KW: Kidney weight
MDA: Malondialdehyde
MPO: Myeloperoxidase
Na+: Sodium
PG12: Prostacyclin
PLA2: Phospholipase
ROS: Reactive oxygen species
Scr: Serum creatinine
TBARS: Thiobarbituricacid reactive substances
TxA2: Thromboxane A2
